# Appropriate Timing and Type of Physical Training in Patients with COVID-19 for Muscle Health and Quality of Life: A Systematic Review

**DOI:** 10.1155/2022/6119593

**Published:** 2022-06-01

**Authors:** I. Gusti Putu Suka Aryana, Siti Setiati, Ivana Beatrice Paulus, Dian Daniella

**Affiliations:** ^1^Division of Geriatrics, Department of Internal Medicine, Faculty of Medicine, Universitas Udayana, Denpasar, Bali, Indonesia; ^2^Division of Geriatrics, Department of Internal Medicine, Faculty of Medicine, Universitas Indonesia, Jakarta, Indonesia; ^3^Wangaya General Hospital, Denpasar, Bali, Indonesia; ^4^Merdeka Medical Center, Denpasar, Bali, Indonesia

## Abstract

Physical activity is beneficial to modulate immune system function and has inverse relationship to ARDS linked with SARS-CoV-2. Physical activity consists of daily activity and physical training. Studies regarding effect of physical training on patients with COVID-19 are controversial. This systematic review aims to investigate physical training on muscle health and QOL in patients with COVID-19. The literature review was carried out using keywords: (Exercise) AND (COVID) AND (Muscle) AND (Observational Study) in several databases of PubMed and Cochrane Central Register of Controlled Trials (CENTRAL). All references were reviewed using critical appraisal Newcastle Ottawa Scale (NOS) and Centre for Evidence-Based Medicine (CEBM) checklist. The studies were subsequently screened for reporting exercise, muscle, and COVID-19. The descriptions of the extracted data are guided by Preferred Reporting Items for Systematic Reviews (PRISMA) statement with GRADE approach. This study is registered in PROSPERO: ID CRD42021295188. Six studies pooled and entered review synthesis. Studies were reviewed using critical appraisal by NOS and CEBM. Two clinical trial studies and four observational designs were selected. Our result showed physical training improved patients' outcomes in the acute phase, critical phase, and post-COVID-19 phase. Multiple types of physical trainings were suggested by those studies, and most of them showed beneficial effects to patients with COVID-19 in different phases. The level of evidence by GRADE was downgraded, and further investigations are needed to establish guidelines and strong recommendation for a specific stage of COVID-19.

## 1. Introduction

At the end of 2019, a pandemic occurred and affected many sectors. Severe acute respiratory syndrome corona virus 2 (SARS-CoV-2) spread globally; on the 11^th^ of March 2020, the World Health Organization (WHO) declared the outbreak a pandemic. On December 6, 2021, the total number of cases confirmed globally was 266,660,144 with 5,266,960 deaths. COVID-19 is characterized by various features from mild to severe. The severe cases may progress to severe clinical manifestations such as acute respiratory distress syndrome (ARDS), septic shock, or even death [1]. Studies found that coronavirus disease (COVID-19) effected muscle in general [2]. COVID-19 weakens respiratory muscles, resulting in gas exchange impairment [3]. This problem persisted 6 months after COVID-19 was resolved [4]. Aschman et al. conducted a study that revealed patients with COVID-19 had significantly more degenerating muscle fibers and inflammation on immunohistochemical analysis and a higher average creatine kinase level [5]. Of the COVID-19 muscle samples, 55% showed upregulation of major histocompatibility complex class 1 (MHC-1) antigens, compared with no control samples [5]. COVID-19 muscle biopsies also had natural killer cells near muscle fibers and myxovirus resistance protein A (MxA) staining in capillaries [5–7]. This further emphasizes the involvement of COVID-19 in muscle weakening.

Muscle is very important as it benefits many organs and tissues, including the immune system [8]. Myokines are proteins, likely cytokine and produced by the muscle itself during physical exercise, which have a role in immunometabolism [8]. Physical activity is beneficial to modulating immune system function and has an inverse relationship to ARDS linked with SARS-CoV-2 [9]. Physical activity consists of activity daily living and physical training [10]. A case reported improved pulmonary function and physical fitness improvement in patients with COVID-19 after a 6-week pulmonary exercise program [11]. The question was raised about the best or optimal timing for physical training and the type of training that should be done in patients with COVID-19.

This systematic review aims to investigate physical training on muscle health and quality of life (QOL) in patients with COVID-19.

## 2. Materials and Methods

### 2.1. Selection of Studies

The eligible publication was screened independently by authors. The inclusion criteria for publications were (1) the whole sample comprising patients with COVID-19; (2) published in English; (3) published since 2019; (4) having randomized clinical trial (RCT) or observational designs. The exclusion criteria were letters, viewpoints, and review studies that provided advice on the topics of muscle and COVID-19.

Generally, studies have focused on the effects of physical training on muscle health and QOL in patients with COVID-19. Muscle health was often defined by the quantity and quality of the muscle, and it was assessed using strength, performance, muscle mass, and respiratory health. Meanwhile, QOL was generally assessed using the 36-Item Short Form Survey (SF-36). This study was registered with PROSPERO (ID CRD42021295188).

### 2.2. Search Strategy

The literature review was conducted using the following keywords: “Exercise” AND “COVID” AND “Muscle” AND “Observational Study.” We conducted the literature search in the following databases: PubMed, Google Scholar, Science Direct, and Cochrane Central Register of Controlled Trials (CENTRAL).

### 2.3. Data Extraction and Assessment

All references were reviewed using the critical assessment tool Newcastle Ottawa Scale (NOS) and the checklist from the Centre for Evidence-Based Medicine. Each author independently reviewed all titles and abstracts of relevant studies. In this first screening, papers were selected based on the established inclusion and exclusion criteria, and the authors checked whether the studies provided reports about exercise, muscle, and COVID-19. Then, the authors independently checked the full-text of papers that passed the initial screening to identify articles relevant to the three aforementioned main topics; duplicate articles were removed at this stage. The following data were collected from the selected studies: authors' names, year of publication, sampling period, study location, sample size, study design, participants' age range, and duration of intervention. Thereafter, each author independently scored and assessed the risk of bias of all included papers, as well as completed a NOS checklist for observational studies and the Cochrane handbook for RCTs. The results were compared, and any controversies surrounding any included or excluded papers were resolved by discussion among the research team. Potentially eligible manuscripts were exported. Data extraction followed the guidelines outlined in the Preferred Reporting Items for Systematic Reviews (PRISMA) statement and checklist. The level of evidence was analyzed based on the Grading of Recommendations Assessment, Development and Evaluation (GRADE) criteria (Table 1) [12].

## 3. Results

**Figure 1:**
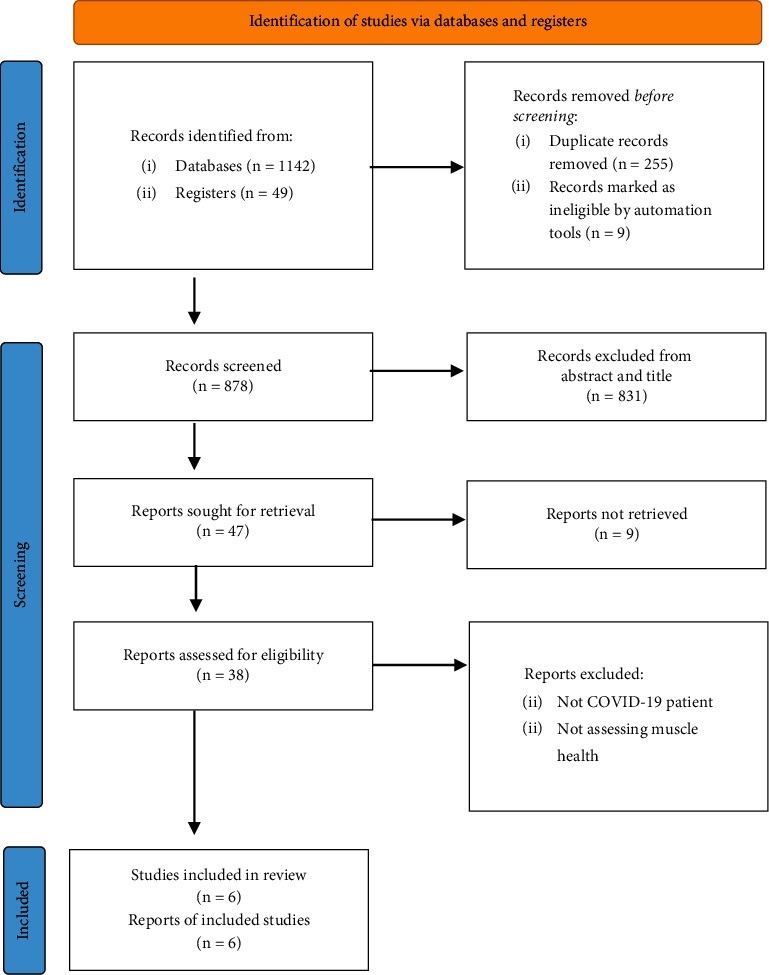
Flowchart of search strategy.

The literature search result and study selection process are presented in Figure 1. Overall, 111 citations of interest were found in the initial electronic searches of PubMed and CENTRAL. After excluding one review, two protocols, 55 duplicate, and six potentially eligible articles were selected. Of these, six full-text papers were potentially relevant and assessed for eligibility. Finally, six papers were included in the systematic review for the evaluated effect of physical training on muscle health and QOL patients with COVID-19, and two clinical trial studies and four observational designs were selected. The selection process algorithm is detailed in Figure 1 and Table 2. Critical appraisals of eligible studies are presented in Table 3. The level of evidence by GRADE was low to high due to the findings being downgraded due to quality (Table 1).

## 4. Discussion

### 4.1. The Effects of Exercise in Patients with COVID-19

#### 4.1.1. Mild COVID-19 Symptoms

In the acute phase of COVID-19, patients with mild symptoms can exercise, but progressive exercise is not recommended in the early stages of acute illness, namely, 48 hours after hospital admission [13]. In hospitalized patients with mild COVID-19 symptoms (median age, 54 years), one month of a modified rehabilitation exercise (MRE) based on the Chinese martial art eight-section Brocade decreased symptom prevalence for dry cough, productive cough, difficulty in expectoration, and dyspnea [13]. MRE is a full-body exercise designed to reduce total airway resistance, smooth fresh airflow, and improve O_2_/CO_2_ exchange efficiency. In the cited study, MRE helped maximize the volume of alveolar sacs and sped up the mucus clearance process, making expectoration easier. Furthermore, when acupressure is incorporated into the MRE, it is believed to relieve COVID-19 symptoms [19]. Although MRE has been proven to be safe and beneficial in alleviating COVID-19 symptoms, RCTs with larger samples are needed to validate the benefits of this type of exercise. In patients with COVID-19, studies showed that acupuncture helps alleviate COVID-19 symptoms [20] modified rehabilitation exercise (MRE) helped minimize COVID-19 symptoms and, thus can be performed in patients with mild COVID-19 symptoms. For patients with mild COVID-19 symptoms, the assigned exercises usually comprise airway clearance, respiratory control, posture management, and physical activity, together with recommendations for a balanced diet and to stop smoking [21]. Generally, there are no activity-related restrictions, so patients can perform the exercise. Furthermore, exercise has a profound impact on the immune system, pulmonary function, and management of COVID-19 [22].

### 4.2. The Effects of Exercise in Patients with COVID-19 Admitted to the ICU

Li et al. conducted a study with 16 patients with COVID-19 in the intensive care unit (ICU) [14] and although 12 were identified as critical, only three received mechanical ventilation (MV). In their study, exercises targeted at the pulmonary system were performed—including body positioning, airway clearance techniques, oscillatory positive end-expiratory pressure, inspiratory muscle training (IMT), and mobility exercises—until ICU discharge, with patients showing a median ICU stay of 15 days. These researchers found that even after the exercise interventions, some patients still had a peak expiratory flow rate (PEFR) and maximum inspiratory pressure (MIP) below 80% of the predicted value, with 46% showing de Morton mobility index values below the normative values [14]. The researchers then remarked that exercise for these patients should be more objectively measured to ensure appropriate training, as each of the used exercises should be performed appropriately if they are to yield better outcomes. Li et al. also reported that training intensity at 50% MIP led to a 20% increase in MIP, whereas a training intensity of 30% MIP demonstrated only an 11% increase. Furthermore, although patients with COVID-19 have impaired respiratory muscle strength, given early rehabilitation program at 50% intensity has been shown to increase respiratory muscle strength in these patients [14].

In a sample of 35 patients with a condition secondary to COVID-19 due to ARDS and who experienced respiratory distress (requiring MV), shock, or organ failure (requiring ICU care), the coupling of an early exercise program (i.e., passive or active range of motion exercises and neuromuscular electrical stimulation, NEMS) with standard intensive care failed to improve handgrip strength [15]. Specifically, the NEMS was applied bilaterally to the quadriceps and tibialis anterior muscles. The exercise program began ≥5 days after ICU stay and ≥10 days after symptom onset, and muscle assessments were performed at discharge from the ICU [15]. Still, this cited study had a small sample size, hindering the ability to draw definite conclusions regarding muscle weakness. Furthermore, the intervention groups had significantly higher rates of chronic pulmonary and neurological diseases, which may have contributed to the nonsignificant results of the interventions. This study also did not perform airway clearance techniques and respiratory muscle training because these interventions have the risk of increased work of breathing in patients [15].

Abodonya et al. found significant improvements in forced vital capacity (FVC), forced expiratory volume in 1 s (FEV1), dyspnea severity index (DSI), and QOL after a 5-day IMT for two weeks in patients with COVID-19 after MV weaning [16]. Particularly, 42 recovered patients with COVID-19 (33 men and 9 women) who weaned from MV in the ICU (mean age, 48 years) were enrolled in the study. The IMT started after MV weaning, and the pulmonary function test, DSI, QOL questionnaire, and functional performance were assessed before the intervention and immediately after the 2-week IMT by a blinded, experienced examiner who was not included in the study intervention [16]. This was the first study to assess the benefits of IMT in patients with COVID-19 after MV weaning. Other research studies also show the benefits of IMT on pulmonary function in patients undergoing hemodialysis [23], severe chronic obstructive pulmonary disease [24], and spinal cord injury [25]. In addition, among patients with previously decreased respiratory muscle function due to immobility caused by MV, IMT helped promote and restore respiratory muscle function. In an older adult sample, IMT helped improve pulmonary function and reduce the time to MV weaning [22]. Hence, it seems that IMT can benefit all populations both before and after MV. Studies that extend the duration of IMT interventions and RCTs with larger samples are warranted to further identify the effect of this exercise in patients with COVID-19 after MV weaning.

In addition to these studies on muscle interventions, some researchers have shown that ICU survivors of COVID-19 may present with severe malnutrition and muscle mass loss. Accordingly, nutritional assessment and early nutritional care management must be integrated into the overall therapeutic strategy for patients with COVID-19 [26]. Additionally, QOL after hospital discharge was shown to be related to psychological factors. Vlake et al. [27] found that 16% of the patients with symptoms suggestive of COVID-19 reported probable post-traumatic stress disorder one month after hospital discharge, and 13% at three months after hospital discharge; for probable anxiety, these numbers were 29% and 20%, respectively; for probable depression, these numbers were 32% and 24%, respectively. These findings show that hospitalization negatively impacts patients' mental health and that they require social support [27].

### 4.3. The Effects of Exercise on Post-COVID-19 Patients

The effects of exercise also extend to post-COVID-19 patients, with these patients being usually assigned to perform aerobic exercises [17]. Li et al. conducted a study with 120 formerly hospitalized COVID-19 survivors with dyspnea complaints (mean age, 50 years), asking them to perform an unsupervised 6-week home exercise program. It comprised 3-4 sessions per week of breathing control, thoracic expansion, aerobic, and lower limb muscle strength exercises. These researchers showed that the exercise program improved patients' performance in the 6-minute walking test and squat time in both the short (6 weeks) and long term (28 weeks) [28]. These results show that the proposed exercise program mainly improved patients' respiratory muscle strength and endurance; nonetheless, the differences in FEV1, FVC, and FEV1/FVC were insignificant, so the program seemingly had a minimal effect on lung volume. Furthermore, although the physical component of QOL showed improvements, there were no improvements in the mental component [27]. This lack of improvements in the mental component may contribute to the return of subjective dyspnea. In another study, exercise programs in post-COVID-19 patients helped improve pulmonary function and QOL in certain aspects, and it was shown that resistance training (especially pulmonary exercise starting in the acute phase) may help reduce post-COVID-19 problems, thereby maximizing its benefits [27, 29, 30].

Sarcopenia is a muscle-related complication of COVID-19 among older adults. Nambi et al. [18] conducted a study with 60–80 years male participants with post-COVID-19 sarcopenia, which was diagnosed by using the appendicular skeletal muscle mass index (score of <7 kg/m^2^ for men was indicative of sarcopenia) [18]. These patients received low-intensity aerobic training (LAT) and high-intensity aerobic training (HAT) for 8 weeks, with one 30-minute session per day for 4 days each week. Resistance training was incorporated into the interventions in both groups. The intensity of exercise was measured using the maximum heart rate and calculated by subtracting 220 from the participants' age. LAT and HAT were defined as 40–60% and 60–80% of maximum heart rate, respectively. LAT improved the clinical (handgrip strength) and psychological (kinesiophobia and QOL) measures more than HAT, albeit there were improvements in both groups [22]. Studies with larger samples and a comparable control group are warranted for providing more in-depth evidence on the potential effects of these exercises on older adults.

Additionally, researchers showed that aerobic exercise helps to prevent a decline in mitochondrial respiration, mitigate aging-related muscle mass loss, and enhance insulin sensitivity [26, 31]. Another study showed that resistance training induces the formation of new satellite cells in weak muscle fibers, the recruitment of new satellite cells for these weak fibers, and increases the number of myonuclei, thereby increasing the strength and power of muscle fibers [28]. Moreover, normal exercise was shown to induce an increase in stroke volume and heart rate, therefore increasing cardiac output; these findings are especially important within the context of older adults because these parameters diminish with aging due to reduced *β*-adrenergic responsiveness, making older adults have lower maximal heart rate, which in turn results in lower exercise capacity [32]. Another study on individuals with frailty used the number of repetitions to demonstrate training intensity, showing that low-intensity exercise (40% repetition) was as effective as high-intensity exercise (70% repetition) with a lower risk of injury, optimal duration of training, and the same fatigue [32]. These findings highlight that low-intensity training is more beneficial than high-intensity training, regardless of the measured variable (for the cited study, maximum heart rate or number of repetitions). To manage sarcopenia, a combination of aerobic and resistance training programs was shown to be the best choice [33]. For optimal muscle function among older people who are malnourished or at risk of malnutrition due to acute or chronic illness, it is suggested to add at least 1.2–1.5 g protein per kg of body weight per day, and these numbers are 1.0–1.2 g for healthy older people [33]. Based on the available evidence and considering that COVID-19 mainly affects the pulmonary system and leads to a higher risk of mechanical ventilation, we suggest that stakeholders should focus more on pulmonary exercises for future interventions in patients with COVID-19 because these are significantly associated with patient outcomes.

This systematic review is the first to assess the effect of exercise on patients with COVID-19. However, there is limited research included in this study, and consequences vary in outcomes, intervention, and study design. The level of evidence by GRADE was downgraded to very low due to the high risk of bias, inconsistency, and uncertainty; therefore, the findings should be interpreted as low quality. In the future, RCT studies regarding the type of exercise in patients with COVID-19 are still needed to provide brief information and improve the quality of review.

### 4.4. Future Directions and Conclusions

Physical training is important for and has shown health benefits when applied to patients with COVID-19, improving various patient outcomes. Specifically, there were beneficial pulmonary outcomes in the acute, critical, and post-COVID-19 phases. Furthermore, the analyzed research proposed multiple types of physical training programs, with most showing beneficial effects in patients with COVID-19 at different phases of the disease. We suggest the addition of physical training to COVID-19 management programs to ensure more holistic management of these patients.

## Figures and Tables

**Table 1:** Grade approach of eligible studies.

Certainty assessment							No. of patients		Effect		Certainty	Importance
No. of studies	Study design	Risk of bias	Inconsistency	Indirectness	Imprecision	Other considerations	Intervention	Comparison	Relative (95% CI)	Absolute (95% CI)		
Mild acute COVID-19 (assessed with self-reported severity of respiratory symptoms development)												
1	Observational studies	Serious^a^	Serious^b^	Not serious	Not serious	None	A total of 60 patients with COVID-19 who completed the full course of MRE were enrolled in this observational study. In total, 60 confirmed mild COVID-19 cases were enrolled with a median age of 54 years old. The baseline prevalence for dry cough, productive cough, difficulty in expectoration, and dyspnea were 41.7%, 43.3%, 35.0%, and 50.0%, respectively				⊕⊕◯◯ Low	Important

*ICU condition (assessed with lung function, handgrip strength, and QOL)*												
3	Observational studies	Not serious^a^	Serious^c^	Not serious	Not serious	Strong association	All patient 93 admitted to ICU was in three studies was given intervention such as a regimented sequence of mobility, early rehabilitation and incentive breathing exercise. After following up the results showed improved of lung function and QOL, however no difference in hand grip strength				⊕⊕◯◯ Low	Important

*Post-COVID-19 (assessed with 6 min walking test (6MWT), lung function, hand grip strength, and QOL questionnaire)*												
2	Randomized trials	Not serious	Not serious	Serious^c^	Not serious	Strong association	All 196 participants post-COVID-19 were involved and received in two studies: 1) study one intervention group: a home-based 6-week exercise programme comprising breathing control and thoracic expansion, aerobic exercise, and LMS exercise; 2) study two received resistance training. The end of the trial show improved lung function, handgrip strength, 6 min walking test (6MWT), and QOL				⊕⊕⊕⊕ High	Important

^a^Small sample study; ^b^Different outcome measurement; ^c^Different intervention.

**Table 2:** Overview of eligible studies.

Author	Design	Setting	Participant	Mean age (years)	Intervention/Physical training	Outcome
Zha et al. [13]	Cohort study	Mild COVID-19	60 patients with mild COVID-19	54 (38–62)	MRE consist of four sets: overhead chest and shoulder stretch (1^st^ set), standing heel raises and upper body acupressure (2^nd^ set), upper body rotation (3^rd^ set), and hand acupressure massage (4^th^ set). The full course of MRE (6–8 repetitions) was done two times a day. Interventions were performed for one month.	Prevalence rate of symptoms decreased from 41.7% to 11.7% in dry cough, 43.3% to 11.7% in productive cough, 35% to 8.3% in difficulty in expectoration, and 50% to 15% in patient-reported dyspnea.

Li et al. [14]	Cohort study	ICU	16 patients with COVID-19 who admitted to ICU	N/A	All 16 patients participated in a regimented sequence of mobility: 10 rolling over and moving on the bed regularly, sitting up in bed, sitting on the bedside, sitting on a chair, standing, and walking (along a 7-m walkway in the ICU) while in ICU.	At discharge from the ICU, 61% and 31% of these patients had PEFR and MIP, respectively, below 80% of the predicted value and 46% had de morton mobility index values below the normative value.

Ozyemisci et al. [15]	Cohort study	ICU	35 patients with ARDS secondary to COVID-19 (18 patients in the rehabilitation group and 17 patients in the nonrehabilitation group)	Rehab 73 (64–78); nonrehab 70 (62–76) (*p*=0.608)	Early rehabilitation program consisting of passive or active ROM exercises and NEMS in addition to standard intensive care compared to standard intensive care. Intervention began ≥5 days of the ICU stay and ≥10 days after the onset of COVID-19 symptoms to patients.	There was no difference in hand grip strength (*p*=1.000) following discharge between rehab and nonrehab groups. No adverse event was noted.

Abodonya et al. [16]	Cohort study	ICU	42 recovered patients with COVID-19 (33 men and 9 women) who were weaned from MV (21 patients in IMT groups and 20 patients in the control group)	IMT group 48.3 ± 8.5; control group 47.8 ± 9.2 (*p*=0.856)	After weaned from MV, each patient was instructed to perform incentive breathing exercise in a relaxed sitting position 2 times daily for 2 following weeks. Each session has consisted of 6 inspiratory cycles; each cycle has remained around 5 min of resisted inspiration, followed by 60-second rest time intending to improve inspiratory muscle strength. At the fifth and sixth cycle, each patient was instructed to breath regularly as much as possible in tending to improve inspiratory muscle fitness.	2 weeks of IMT improves pulmonary functions (FVC; *p*=0.041 and FEV1; *p*=0.043), dyspnea (DSI; *p*=0.032), functional performance (6MWD; *p*=0.028), and QOL (*p*=0.021) compared to the control group.

Li et al. [17]	RCT	Post-COVID-19 hospital discharge	120 COVID-19 survivors with remaining dyspnea complaints (59 patients in the TERECO group and 61 patients in the control group)	Intervention 49.17 (10.75); control 52.03 (11.10)	Unsupervised home-based 6-week exercise programme comprising breathing control and thoracic expansion, aerobic exercise and LMS exercise, delivered via smartphone, and remotely monitored with heart rate telemetry. Outcome was assessed post-treatment (6 weeks) and followed up again in after 28 weeks.	Between-group difference in mean change of 6MWT was 65.45 m (*p* < 0.001) at post-treatment and 68.62 m (*p* < 0.001) at follow-up and squat time was 20.12 m (*p* < 0.001) at post-treatment and 22.23 (*p* < 0.001) at follow-up. Insignificant differences were noted from FEV1, FVC and FEV1/FVC (*p* > 0.05) Increase in SF-12 physical component was greater in the TERECO group with treatment effects estimated as 3.79 (*p*=0.004) at post-treatment and 2.69 (*p*=0.045) at follow-up.

Nambi et al. [18]	RCT	Post COVID-19	76 men in 60–80 years with post-COVID-19 and sarcopenia (38 men in the LAT group and 38 men in the HAT group)	LAT 63.2 ± 3.1; HAT: 4.1 ± 3.2 (*p* 0.217)	All participants received resistance training for whatever time of the day that they received it, and that in addition they were randomized into two LAT (40–60% of maximum heart rate) and HAT (60–80% of maximum heart rate) groups for 30 minutes/session, 1 session/day, 4 days/week for 8 weeks.	At the end of six-months follow-up, the handgrip strength (−3.9), kinesiophobia level (4.7), and QOL (−10.4) shows more improvement (*p* < 0.001) in the LAT group than the HAT group.

ICU: intensive care unit; ARDS: acute respiratory distress syndrome; RCT: randomized controlled trial; NEMS: neuromuscular electrical stimulation; ROM: range of motion; N/A: not available; IMT: inspiratory muscle training; FEV1: forced expiratory volume in 1 second; FVC: forced vital capacity; DSI: dyspnea severity index; COVID-19: coronavirus disease-19; MV: mechanical ventilation; QOL: quality of life; MRE: modified rehabilitation exercise; 6MWD: 6-minute walking distance; LMS: lower limb muscle strength; TERECO: telerehabilitation programme in postdischarge patients with COVID-19; LAT: low-intensity aerobic training; HAT: high-intensity aerobic training.

**Table 3:** Critical appraisal of eligible studies.

Author	Design	Selection	Comparability	Outcome
Zha et al. [13]	Cohort study	^*∗∗∗*^	^*∗*^	^*∗∗*^
Li et al. [14]	Cohort study	^*∗∗*^		^*∗∗∗*^
Ozyemisci et al. [15]	Cohort study	^*∗∗∗∗*^	^*∗*^	^*∗∗∗*^
Abodonya et al. [16]	Cohort study	^*∗∗∗∗*^	^*∗*^	^*∗∗∗*^
		Validity	Importance	Applicability
Li et al. [17]	RCT	(+)	(+)	(+)
Nambi et al. [18]	RCT	(+)	(+)	(+)

RCT: randomized controlled trial.

## Data Availability

The data used to support the findings of this study are fully available in the manuscript body.

## Supplementary Materials

Supplementary Materials The section shows the tabular column of Prisma 2020 checklist.
